# Recurrence and histological evolution of dysembryoplastic neuroepithelial tumor: A case report and review of the literature

**DOI:** 10.3892/ol.2013.1480

**Published:** 2013-07-22

**Authors:** LV CHAO, XU BO TAO, YANG KAI JUN, HAN HUI XIA, WANG KE WAN, QI SONG TAO

**Affiliations:** 1 Department of Neurosurgery, Nan Fang Hospital, Southern Medical University, Guangzhou, Guangdong 510515, P.R. China; 2 Department of Pathology, Nan Fang Hospital, Southern Medical University, Guangzhou, Guangdong 510515, P.R. China

**Keywords:** dysembryoplastic neuroepithelial tumor, astrocytoma, recurrent, malignant transformation, prognosis

## Abstract

Studies of recurrent dysembryoplastic neuroepithelial tumors (DNTs) are distinctly rare. The present study reports the case of a 15-year-old female with a temporal lobe DNT, which recurred and transformed into an astrocytoma (WHO grade II) five years after an initial gross total resection (GTR). Furthermore, all the previous studies on recurrent DNT are reviewed. Although the majority of DNT cases demonstrate benign behavior, recurrent DNTs have been observed following a GTR of the tumor. Patients do not appear to benefit from post-operative adjuvant therapy, and inappropriate radiotherapy or chemotherapy may result in tumor recurrence or malignant transformation. The prognosis is favorable if a GTR of the recurrent tumor is achieved. The use of regular imaging examinations and the maintenance of a long-term follow-up is of importance following a tumor resection.

## Introduction

A dysembryoplastic neuroepithelial tumor (DNT) is widely recognized as a benign lesion and is classified as a neuronal and mixed neuronal-glial tumor, corresponding to WHO Grade I (1). DNTs have been shown to correlate with intractable epilepsy and are usually located in the supratentorial cortex (2–4).

DNTs are considered curable with surgery alone, without the use of adjuvant therapy (5–7). However, studies have indicated certain instances of tumor recurrence, with the majority of tumors recurring secondary to an initial subtotal resection (STR) or partial resection surgery (8–10). Certain cases have been shown to exhibit recurrence following a gross total resection (GTR) (11–14). Rare cases have reported tumors that progressed to high-grade astrocytomas (8,9,14–17), certain cases of which may have been the result of inappropriate post-operative radiotherapy (14–15). The present study reports a histological evolution DNT case. The patient underwent a standard anterior temporal lobectomy in the Department of Neurosurgery, Nan Fang Hospital (Guangzhou, China) and a microscopic pathological evaluation demonstrated that the lesion was a DNT. The patient was administered adjuvant chemotherapy in another hospital at one month post-surgery. The tumor recurred *in situ* five years after the initial surgery. Microscopic pathological evaluation disclosed fibrillary astrocytoma (WHO grade II) as the predominant component of the recurrent tumor. Written informed consent was obtained from the patient.

## Case report

**Figure 1:**
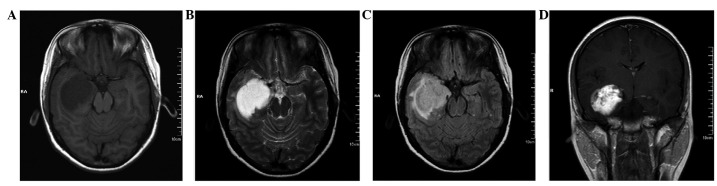
Pre-operative MRI of the initial surgery. (A) A hypointense well-defined lesion in the right temporal lobe, shown by T1WI. (B) A homogeneous hyperintense lesio, shown by T2WI. (C) A heterogeneous hyperintense lesion with slight edema, shown by FLAIR sequence imaging. (D) Patchy enhancement of the tumor, shown by contrast-enhanced imaging. MRI, magnetic resonance imaging; WI, weighted imaging; FLAIR, fluid-attenuated inversion recovery.

In October 2006, a 15-year old female with a three-week history of partial complex seizures and an unusual saline taste as the aura before seizure attack was admitted to the Department of Neurosurgery, Nan Fang Hospital. Magnetic resonance imaging (MRI) revealed a well-defined lesion in the right temporal lobe that was 3.1×4.3×6.4 cm in size. No obvious peritumoral edema or mass effect was observed. T1 weighted imaging (WI) revealed the lesion to be hypointense (Fig. 1A), while T2WI showed it to be hyperintense (Fig. 1B). Fluid-attenuated inversion recovery (FLAIR) sequence imaging displayed the lesion as heterogeneously hyperintense (Fig. 1C). Contrast-enhanced imaging revealed a patchy enhancement of the tumor (Fig. 1D). During the interictal period, a scalp EEG demonstrated α-waves with a frequency of 9–10 Hz and an amplitude of 40–100 μV, mainly as the background rhythm. A distribution of high amplitude sharp waves, spikes and sharp-slow waves were observed in the lesion area.

**Figure 2:**
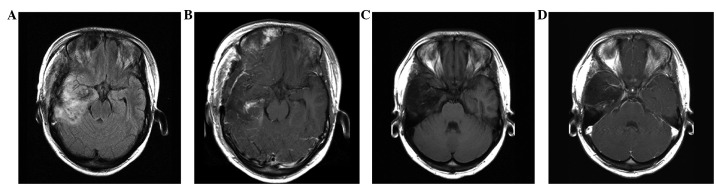
MRI following the initial surgery at (A and B) three days post-surgery and (C and D) one year post-surgery. (A) Slight brain edema in the surgical region (FLAIR sequence imaging). (B) Funicular enhancement, which may have been due to slight hemorrhaging of the brain (contrast-enhanced imaging). (C) T1WI with recurrent manifestation. (D) Contrast-enhanced imaging without any obvious enhancement. MRI, magnetic resonance imaging; WI, weighted imaging; FLAIR, fluid-attenuated inversion recovery.

The tumor was located in the cortex with a dim appearance and moderate blood supply. A clear boundary and no capsule was observed, as determined by pre-operative MRI. The lesion, ipsilateral anterior temporal lobe, hippocampus and amygdala were removed by a standard right anterior temporal lobectomy and a GTR was achieved (Fig. 2).

**Figure 3:**
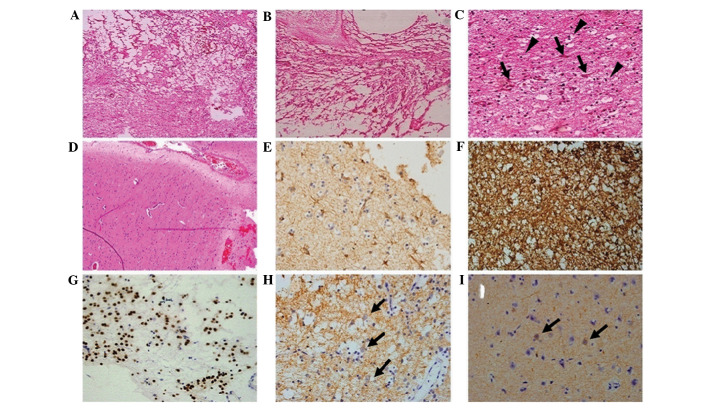
Pathology data from the first surgery. (A and B) A typical loose reticular degeneration with microcapsule formation and matrix mucoid degeneration (HE staining; magnification, ×4). (C) Hyperplastic oligodendrocyte-like cells (black triangles) and immature neurons (black arrows) in the tumor section (HE staining; magnification, ×40). (D) Evidence of FCD in the peritumoral cortex (HE staining; magnification, ×20). (E) Astrocytoma, (GFAP staining; magnification, ×40). (F) Hyperplastic gliacyte component, including oligodendrocyte-like cells and astrocytoma (S-100 staining; magnification, ×40). (G) Oligodendrocyte-like cells, (Oligo-2 staining; magnification, ×40). (H) Immature neurons (black arrows; Syn staining; magnification, ×40). (I) Immature neurons (black arrows; NF staining; magnification, ×40). HE, hematoxylin and eosin; FCD, focal cortical dysplasia; GFAP, glial fibrillary acidic protein; Syn, synaptophysin; NF, neurofilament.

The microscopic evaluation revealed a typical form of DNT (Fig. 3A and B), which was composed of a specific glioneuronal element with floating neurons within small mucoid lakes (Fig. 3C). Glial cell proliferation was notable, including numerous oligodendrocyte-like cells and fewer astrocytes. The oligodendrocyte-like cells shared the same appearance in a diffuse manner with rare mitotic figures. In addition, focal cortical dysplasia (FCD) was identified in the peritumoral cortex (Fig. 3D). The immunohistochemical results demonstrated that glial fibrillary acidic protein (GFAP) and S-100 were positive in the astrocytes and oligodendrocyte-like cells (Fig. 3E and F). Oligo-2 was positive in the majority of the oligodendrocyte-like cells (Fig. 3G). The immature neurons were positive for synaptophysin (Syn; Fig. 3H) and neurofilament (NF; Fig. 3I). The pathological diagnosis of the tumor was of a DNT, WHO grade I. The post-operative course was uneventful and the seizures were controlled using an oral antiepileptic. The dose of the drug was gradually reduced over two years following the surgery. At one month post-surgery, the patient was administered adjuvant chemotherapy with temozolomide in another hospital. The regimen was recorded as 150 mg/m^2^/day, orally, once a day on 5 consecutive days, for 28 days.

**Figure 4:**
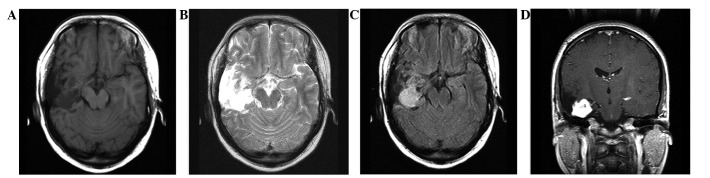
MRI of the recurrent tumor. (A) T1WI indicating tumor recurrence *in situ*, with a homogeneous and hypointense signal. (B) T2WI indicating a hyperintense lesion. (C) No obvious peripheral edema on FLAIR sequence imaging. (D) Intense enhancement of the recurrent lesion, shown by contrast-enhanced imaging. MRI, magnetic resonance imaging; WI, weighted imaging; FLAIR, fluid attenuated inversion recovery.

Five years after the initial surgery, the patient reported an intermittent headache. A neurological examination did not reveal any abnormalities. However, MRI revealed that a new lesion had occurred at the base of the previous surgery, with a size of 2.2×2.0×1.8 cm. The lesion was hypointense on T1WI (Fig 4A) and hyperintense on T2WI (Fig. 4B). No edema was observed on the FLAIR sequence imaging (Fig. 4C). Contrast-enhanced imaging revealed intense enhancement of the tumor (Fig. 4D).

**Figure 5:**
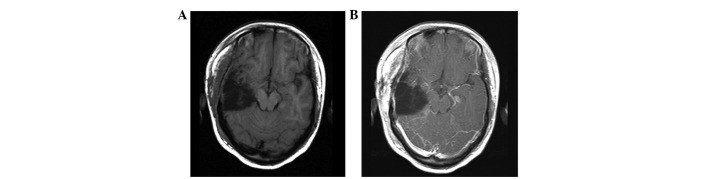
MRI three days after the second surgery. (A) No residual tumor, shown by T1WI. (B) No obvious enhancement manifestation on the contrast-enhanced imaging. MRI, magnetic resonance imaging; WI, weighted imaging.

In October 2011, the patient underwent a second surgery using the same route as for the previous procedure. The tumor originated from the insular lobe and was a tenacious mass with marked vascularity and a grey-colored appearance. GTR was achieved (Fig. 5) and the post-operative course was uneventful.

**Figure 6:**
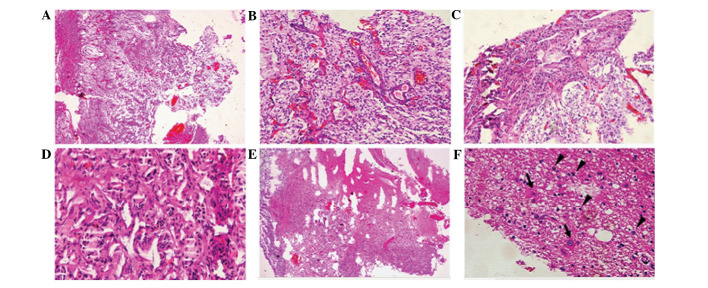
Pathology data from the second surgery. (A) Recurrent tumor tissue (HE staining; magnification, ×4). (B) Diffuse distribution of the tumor cells with pale cytoplasm, polymorphous nuclei and small vessel proliferation (HE staining; magnification, ×20). (C) The fibrous arrangement of the tumor cells with marked hyperchromatism and pleomorphism (HE staining; magnification, ×20). (D) Epithelioid-like proliferation of tumor cells (HE staining; magnification, ×40). (E) A typical loose reticular degeneration with microcapsule formation in a section of the recurrent tumor (HE staining; magnification, ×4). (F) Immature neurons (black arrows) and oligodendrocyte-like cells (black triangles) in the tumor resection (HE staining; magnification, ×40). HE, hematoxylin and eosin.

**Figure 7:**
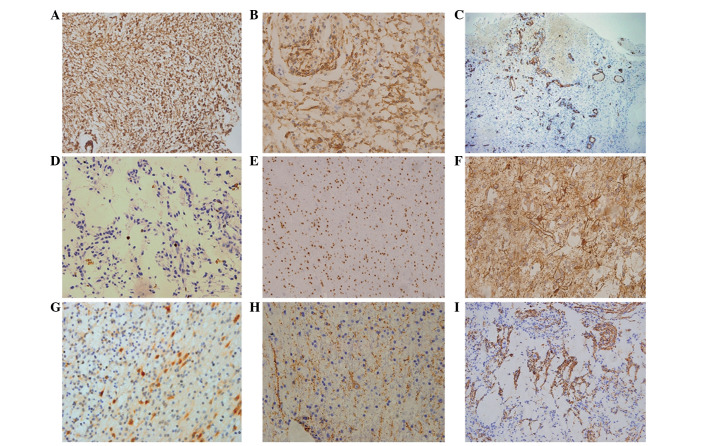
Pathology data following the second surgery. (A) Hyperplastic gliacyte component (GFAP staining; magnification, ×20). (B) Hyperplastic gliacyte component (S-100 staining; magnification, ×40). (C) Hyperplasia of the capillaries (CD34 staining; magnification, ×4). (D) Rare mitotic figures of the tumor (Ki-67 staining; magnification, ×40). (E) Oligodendrocyte-like cells in the region of the microcapsules (Oligo-2 staining; magnification, ×20). (F) Astrocytoma in the region of microcapsules (GFAP staining; magnification, ×40). (G) Immature neurons (NeuN staining; magnification, ×40). (H) Immature neurons (Syn staining; magnification, ×40). (I) The focal positive reaction of the tumor (NF staining; magnification, ×20). GFAP, glial fibrillary acidic protein; Syn, synaptophysin; NF, neurofilament.

The microscopic evaluation of the recurrent lesion disclosed two distinct morphological patterns. The prevailing area revealed the typical appearance of a fibrillary astrocytoma (Fig. 6A). The tumor cells were diffusely distributed with a pale cytoplasm and polymorphous nuclei (Fig. 6B) and mitotic activity was absent. The tumor underwent small vessel proliferation (Fig. 6B), which may have accounted for the results of the contrast-enhanced imaging. Certain tumor cells exhibited a fibrous arrangement (Fig. 6C) or epithelioid-like proliferation (Fig. 6D), in which marked hyperchromatism and pleomorphism were observed. The secondary component was identified only in a small section of the tumor (<20% of the total tumor bulk) and shared similar pathological features with the initial tumor that was identified in 2006 (Fig. 6E and F). However, the oligodendrocyte-like cells in this area were pleomorphic and binucleated and multinucleated cells were visible (Fig. 6F). The prevailing section of the recurrent tumor revealed the strong expression of GFAP and S-100 (Fig. 7A and B). The endothelium of the hyperplastic vessel was positive for CD-34 (Fig. 7C) and the Ki-67 index was <3% (Fig. 7D). In the secondary section of the tumor, only the oligodendrocyte-like cells were positive for Oligo-2 (Fig. 7E), while the astrocytes and the oligodendrocyte-like cells were positive for GFAP (Fig. 7F). The immature neurons expressed NeuN, Syn and NF (Fig. 7G and I). The final pathological diagnosis was fibrillary astrocytoma, WHO grade II.

Adjuvant radiotherapy was administered post-operatively. The radiation dose in the tumor region was 54 Gy/27 F and 48 Gy/27 F in the edema region. During an 11-month follow-up period, the patient was in good condition without any neurological disorder. No recurrence or residual tumor was identified using MRI.

## Discussion

First reported in 1988 (18), DNT was classified as a neuronal and mixed neuronal-glial tumor in 2000 (1). The correlation between DNT and intractable epilepsy has been widely recognized and epilepsy caused by DNT may account for 0.8–6.8% of all intractable epilepsy cases (1,16,19–21). In patients with DNT, seizures are not controlled by an oral antiepileptic drug and surgical treatment is the most effective method of management. Usually, post-operative adjuvant treatment is unnecessary, as the tumor seldom recurs following a GTR. According to the literature, the majority of STR cases share a similar prognosis to GTR cases (6,11,5,16).

Since the initial study of a recurrent case in 2000 (9), 36 similar cases have been reported, including the present study. All the reported cases of recurrent DNT are listed in Table I, in which three cases of radiographic progression are included. These cases have shown that DNT has a wider spectrum of clinical behaviors than those that were initially reported by Daumas-Duport *et al*(18). As a brain tumor corresponding to WHO Grade I, DNT retains the potential for recurrence and malignant transformation.

The excessive growth of any component of DNT, including immature neurons, oligodendrocyte-like cells and astrocytes, may lead to tumor recurrence or post-operative malignant transformation. The present case of recurrence may be attributed to the administration of inappropriate adjuvant chemotherapy following surgery. The patient is also the first histological evolution case that may have been caused by chemotherapy alone. Based on the current treatment guidelines for a low-grade brain tumor, adjuvant therapy is unnecessary for a WHO grade I tumor. However, the patient was administered chemotherapy with temozolomide in another hospital and the tumor recurrence was considered to be directly associated with this inappropriate chemotherapy. A further two similar cases have been identified in previous studies, cases 2 (15) and 26 (14), in which the patients underwent an STR of the tumor prior to 1988, when little was known about DNT. Case two was diagnosed as a fibrillary astrocytoma at the initial surgery, after which, the patient was administered adjuvant radiotherapy and chemotherapy. The patient from case 26 was administered adjuvant radiotherapy for the initial diagnosis of a protoplasmic astrocytoma. At that time, adjuvant therapy for residual tumors of WHO grade II was acceptable. In the follow-up period, the tumors recurred and progressed into astrocytoma, WHO grade III, at 72 months (case 2) and 80 months (case 26).

The present case had a typical imaging manifestation shown by T1WI, T2WI and FLAIR sequence imaging, as described in previous studies of DNT. However, contrast-enhanced imaging revealed the tumor with rare, patchy enhancement. Although the tumor was initially diagnosed as a pilocytic astrocytoma or pleomorphic xanthoastrocytoma based on the manifestation on the neuroimaging, microscopic pathology revealed the tumor to be a typical DNT. From a review of the previous literature, similar presentations have been identified using enhanced imaging in a number of cases (11,12,22,30,31).

In the 36 reported cases, including the present study, 20 cases exhibited clear pathological evidence that demonstrated the tumor recurrence or malignant transformation. The average tumor-free survival time was 65.3 months, (range, 10–132 months). This survival time was similar to that reported by Ray *et al*(14). The extent of the resection following the initial surgery was available in 16 cases, of which, 10 resulted in an STR compared with six GTRs. The average tumor-free survival time of the STR and GTR groups was 61.1 months (range, 12–132 months) and 66.3 months (range, 23–88 months), respectively. No statistical difference was observed between the two groups based on the independent-samples t-test (P=0.757).

Of the 17 cases in which the exact pathological diagnosis of the recurrent tumor was available (the three patients who were administered adjuvant radiotherapy and chemotherapy were excluded from this comparison), DNT recurrence without malignant transformation was demonstrated in 11 cases and the remaining six cases represented malignant transformation or histological evolution. The average tumor-free survival time of the recurrence group was 64.9 months (range, 10–125 months) compared with an average tumor-free survival time of 69.3 months (range, 24–132 months) in the malignant-transformation group. No statistical difference was observed between the two groups based on the results of the independent-samples t-test (P=0.818).

In all recurrent cases with a pathological diagnosis, the average recurrence time of those who were administered adjuvant therapy was 58.7 months (range, 36–80 months) compared with 69.3 months (range, 24–132 months) for the patients who only underwent surgery. No statistical difference was observed between the two groups based on the independent-samples t-test (P=0.684). Therefore, this may be evidence that post-operative adjuvant therapy for DNT is not able to prolong the tumor-free survival time and benefit patients. The reason for tumor recurrence in the three patients who were administered adjuvant therapy is unknown. Hammond *et al*(9) and Ray *et al*(14) hypothesized that radiotherapy was a risk factor for tumor recurrence, and the present study may indicate that chemotherapy is another risk factor. Notably, malignant tumor transformation without any adjuvant therapy has been reported in six cases and remains unexplained. The recurrence and malignant transformation of DNT may have occurred due to the innate potential of the tumor itself, and the adjuvant radiotherapy or chemotherapy may have initiated the key steps towards malignant transformation.

To date, no treatment guidelines for recurrent DNT have been available. Usually, a second surgery is performed in cases of tumor recurrence without malignant transformation (7,12–14). For malignant transformation or histological evolution cases, there are no fixed views on treatment. We prefer a comprehensive treatment, including surgery and post-operative adjuvant therapy. Ray *et al*(14) reported a patient whose tumor recurred and progressed into an astrocytoma (WHO grade III). The patient was administered adjuvant chemotherapy with temozolomide and showed a favorable post-operative prognosis. A similar case has been reported in which the patient underwent a GTR of the recurrent tumor without any adjuvant therapy (16). In the majority of patients in whom the recurrent tumor has progressed into a WHO Grade II tumor, a favorable prognosis may be achieved through a GTR of the recurrent lesion. The case of a patient who remained tumor-free five years following the second surgery has also been reported (10,17,22,23). In the present case, the patient underwent focal radiotherapy and the tumor did not recur within a follow-up period of 11 months.

In conclusion, as a WHO Grade I tumor, DNT retains the potential for recurrence and malignant transformation following GTR. Post-operative adjuvant therapy is not able to prolong the tumor-free survival time and may be a risk factor for tumor recurrence. For recurrent cases, the prognosis is favorable if a GTR of the recurrent lesion is achieved. Based on the previous evidence, adjuvant therapy is not recommended for a definitive diagnosis of DNT. The use of regular imaging examinations and the maintenance of a long-term follow-up is of importance following a tumor resection.

## Figures and Tables

**Table I:** Summary of the previous and current reports of recurrent or progressive DNTs.

First author, year (ref)	Case no.	Age at first resection (years)/gender	Initial pathological diagnosis	Location	Resection	Adjuvant therapy	Interval between first and second resection	Recurrence/progression
Hammond *et al,* 2000 (9) and Duggal *et al,* 2008 (8)	1	29/M	Fibrillary astrocytoma (1984), rediagnosed as DNT (1995)	Left frontal lobe	STR	None	11 years	Astrocytoma, WHO grade IV
Rushing *et al*, 2003 (15)	2	14/M	Mixed low-grade oligoastrocytoma (1974), rediagnosed as DNT (2003)	Right temporo- paritetal lobe	STR	Radiotherapy, chemotherapy	3 years	DNT plus anaplastic astrocytoma plus radiation changes
Fernandez *et al*, 2003 (11)	3	6/M	DNT	Frontal lobe	GTR	None	125 months	Pathological diagnosis unknown
Nolan *et al*, 2004 (5)	4	Unknown	DNT	Unknown	STR	None	≤12 months	Radiographic progression
	5	Unknown	DNT	Unknown	STR	None	≤12 months	Radiographic progression
	6	Unknown	DNT	Unknown	STR	None	≤12 months	Radiographic progression
Sakuta *et al*, 2005 (7)	7	8/M	DNT	Left parietal lobe	STR	None	6 years	Recurrence
	8	4/F	DNT	Left parietal lobe	STR	None	1 year	Recurrence
	9	10/M	DNT	Right temporal lobe	GTR	None	6.9 years	Recurrence
Jensen *et al*, 2006 (12)	10	29 (MRI)/F	-	Temporal lobe	No initial resection radiographic progression	None	15 years	DNT (first MRI to but surgery)
Josan *et al*, 2007 (19)	11	3 (MRI)/unknown	-	Parietal lobe	STR	None	11 years	Radiographic progression without initial pathological diagnosis
Gonzales *et al*, 2007 (23)	12	34/M	DNT	Left temporal lobe	Not stated	Unknown	125 months	Recurrence
	13	32/F	DNT	Right frontal lobe	Not stated	Unknown	98 months	Recurrence
	14	33/F	DNT	Right amygdala	Not stated	Unknown	64 months	Recurrence
	15	47/F	DNT	Left frontal lobe	STR	None	40 months	DNT plus oligoastrocytoma, WHO grade II
Schittenhelm *et al,* 2007 (10)	16	7/F	DNT	Mutifocal lesions	STR	None	7 years	DNT with atypia and Ki-67 index up to 10%
Maher *et al,* 2008 (13)	17	6/M	DNT	Temporo-occipital lobe	GTR	None	6 years	Recurrence
Minkin *et al*, 2008 (24)	18	Unknown	DNT and pilocytic astrocytomas	Unknown	GTR	None	Unknown	No sign of progression as stated
Sung and Suh, 2009 (25)	19	16/M	DNT	Occipital lobe	Not stated	Not stated	Not stated	Not stated
	20	64/F	DNT	Occipital lobe	Not stated	Not stated	Not stated	Not stated
Lee *et al*, 2009 (26)	21	16/M	DNT	Occipital lobe	Lesionectomy	None	5 years	No sign of progression as stated
Ray *et al*, 2009 (14)	22	20/F	DNT	Right frontal lobe	GTR	None	72 months	Recurrence
	23	37/M	DNT	Left temporal lobe	STR	None	83 months	Recurrence
	24	16/M	DNT	Left temporo- occipital lobe	GTR	None	88 months	Pilocytic astrocytoma, WHO grade I
	25	18/M	DNT	Right parietal lobe	GTR	None	23 months	Recurrence
	26	12/M	Protoplasmic astrocytoma, rediagnosed as DNT	Right fronto-parietal lobe	STR	Radiotherapy	80 months	Astrocytoma, WHO grade III
Zakrzewski *et al*, 2009 (17)	27	7/F	DNT	Right temporal lobe	STR	None	4 years	Pilocytic astrocyoma, WHO grade I
Kawataki *et al*, 2010 (27)	28	1 (MRI)/M	-	Left frontal lobe	No initial resection, but radiographic progression	None	7 years (first CT to surgery)	DNT
Qaddoumi *et al*, 2010 (28)	29	6/unknown	DNT	Temporal lobe	STR	None	12 years	No sign of progression as stated
	30	9/unknown	DNT	Temporal lobe	GTR	None	Unknown	Unknown
	31	9/unknown	DNT	Temporal lobe	GTR	None	Unknown	Unknown
	32	13/unknown	DNT	Temporal-parietal- occipital lobe	GTR	None	Unknown	Unknown
	33	11/unknown	DNT	Temporal lobe	GTR	None	Unknown	Unknown
Thom *et al*, 2011 (16)	34	56/unknown	DNT	Left temporal lobe	STR	None	2 years	Anaplastic mixed glioneuronal tumor, WHO grade III
Wagner *et al*, 2012 (29)	35	9/unknown	DNT	Unknown	Unknown	Unknown	10 months	Recurrence
Present case study	36	15/F	DNT	Right temporal lobe	GTR	Chemotherapy	5 years	Astrocyoma, WHO grade II

GTR, gross total resection; STR, subtotal resection; WHO, World Health Organization; Unknown, information not recorded; M, male; F, female; DNT, dysembryoplastic neuroepithelial tumor; MRI, magnetic resonance imaging; CT, computed tomography.

## Abbreviations

DNT: dysembryoplastic neuroepithelial tumors
WI: weighted imaging
FCD: focal cortical dysplasia
Syn: synaptophysin
GTR: gross total resection
STR: subtotal resection
